# Defining quality assessment in vascular surgery training: an expert Delphi process

**DOI:** 10.1308/rcsann.2022.0146

**Published:** 2023-02-13

**Authors:** HC Travers, AJ Beamish, M McCarthy, DR Lewis

**Affiliations:** ^1^ Russell’s Hall Hospital, UK; ^2^ Vascular Surgery Specialty Advisory Committee, UK; ^3^ Swansea University Medical School, Swansea University, UK; ^4^ University Hospitals of Leicester, UK; ^5^ Aneurin Bevan University Health Board, UK

**Keywords:** Delphi, Medical education, Surgical training, Vascular surgery

## Abstract

**Introduction:**

A robust and reproducible way of assessing training should optimise and standardise vascular surgical training. This study describes the methodology supporting the Vascular Surgery Specialty Advisory Committee regional quality assurance reports for vascular surgery training programmes in the UK.

**Methods:**

A Delphi consensus exercise was performed to establish the domains of training that most appropriately assess the quality of a vascular surgery training programme. A total of 54 stakeholders were invited to participate, including trainees, training programme directors and members of the vascular speciality advisory committee (SAC), vascular society executive and education committees.

**Results:**

A total of 39 stakeholders successfully completed the three-stage Delphi process over 15 weeks. The domains identified as most appropriate to assess the quality of a vascular training programme were: Joint Committee on Surgical Training (JCST) survey results, clinical experience, regional education programmes, radiology support, timetable, regional support for trainees, trainer support for trainees, opportunities for professional development, trainee-rated quality of consultant teaching and training, and trainee recommendation of the post.

**Conclusions:**

This study describes a method to identify and prioritise domains that are appropriate to assess the quality of a vascular training programme. The domains that were identified as appropriate to assess quality are transferable internationally and the Delphi methodology could be used by other training schemes to ‘fine-tune’ their own domains to review and optimise the quality of their own training programmes.

## Introduction

On 16 March 2012, an act of Parliament was passed and vascular surgery was designated a stand-alone surgical specialty of the General Medical Council (GMC).^1,2^ The Joint Committee for Surgical Training (JCST) assembled a new Vascular surgery Specialty Advisory Committee (SAC) to design and deliver a new curriculum. In 2019, the training programme delivered its first cohort of fully trained vascular specialists who had completed the new curriculum.^3^

Quality assurance (QA) in training programmes is imperative to ensure the delivery of safe, competent, specialist doctors.^4^ For vascular surgery in the UK, QA is the responsibility of the vascular SAC, through its QA Group, under the oversight of JCST. The vascular SAC comprises consultant vascular surgeons with an interest in medical education from across the UK and Ireland, along with a trainee representative. Each of the UK’s 13 deaneries and The Republic of Ireland has a committee member, known as the Liaison Member (LM), who acts as the link between the Deanery Training Programme Director (TPD) and the SAC.^5^

Historically, the SAC QA group collated annual reports on the vascular training programme in each Deanery. Information contributed by TPDs and LMs, together with the results of an annual end-of-year trainee survey conducted by the vascular trainee representative body (the Rouleaux Club), is used. Data from these three sources were collated and a formal specialty report compiled, outlining the Deanery-specific training programme quality based subjectively on the information provided with no guidance or scoring system.

This QA process was subjective and susceptible to a lack of continuity, because the QA Lead changes regularly and the trainee representative demits every 2 years. As such, a more objective, transparent and reproducible measure of the quality of training programmes was needed.

The JCST has a QA strategy, which it delivers and monitors via the SAC, and in 2018 the JCST committed to produce annual regional quality reports to improve the accessibility of QA information regarding local surgical training programmes. In order for the Vascular SAC to achieve this, specialty-specific quality indicators were needed. This study aimed to determine appropriate training programme quality indicator domains to inform future QA processes and the development of a scoring system to assess quality in the UK vascular surgery training programme.

## Methods

### Study design and participants

A three-stage modified Delphi consensus process was completed to establish general domains on which to assess the quality of a vascular training programme.

### Pre-Delphi: identify potential domains and experts

Authors and members of the QA Group (SAC trainee representative, SAC chair and SAC QA lead) compiled a list of potential domains using all sources previously used in training programme QA. These included JCST quality indicators, JCST trainee survey, GMC survey, LM reports and TPD reports (Table 1).

**Table 1:** Possible domains

**Domain**	**Factors to be considered in the domain**
JCST survey results	
GMC survey results	
Clinical experience	Operative experience, outpatient sessions, MDT, ward rounds
Subspecialty training opportunities	
Educational programmes	Frequency and quality of local departmental teaching, Regional/ Deanery teaching, Journal club, X-ray meetings with educational component, MDTs with an educational component, Attendance at ASPIRE courses
Trainer support of trainees	OOH supervision, clinic supervision, operative supervision
Radiology support	Access to IR training lists, enthusiasm to train
Opportunities for professional development	Access to study leave for courses and conferences, attendance at courses and conferences
Access to and provision of simulation training	Attendance at ASPIRE courses, local provision of high-fidelity simulation, NOTSS and human factors training
Research opportunities and output	Proportion of trainees with higher degrees, output
ARCP outcomes	
Training post information and administration	Vascular only rota from ST5 onwards, number of trainees in a unit, relevant general surgical firms, geographical location of training post/size of deanery, administration of training programme and training posts, e.g. knowing placements/rotas in a timely fashion
Regional support for trainees	Opportunity and support for LTFT if required; opportunity and support for parental leave; experienced episodes of being bullied, harassed or undermined; witnessed episodes of bullying, harassment or undermining; professional advice and support SuppoRTT programme for all those returning to training
FRCS (Vasc) exam results	Attempts and pass rate Part 1/Part 2
WBA	Number of WBA completed; length of time between performing WBA and it being entered on ISCP; support and engagement of trainers with ISCP
Timetable	Sessions range and type; number of clinics; number of theatre lists; vascular only rota
Completion of checklists	ST4/6/8
Trainee recommendation of the post as per JCST survey	
Trainee rated quality of consultant teaching and training as per JCST survey	
Quality of AES feedback report	

AES = assigned educational supervisor; ARCP = annual review of competence progression; ASPIRE = Annual Specialist Registrar Educational; FRCS = Fellowship of the Royal Colleges of Surgeons; GMC = General Medical Council; IR = interventional radiologists; ISCP = Intercollegiate Surgical Curriculum Programme; JCST = Joint Committee for Surgical Training; LTFT = less than full time; MDT = multidisciplinary team; NOTSS = nontechnical skills for surgeons; OOH = out-of-hours; SuppoRTT = supported return to training; WBA = work based assessments

**Figure 1:**
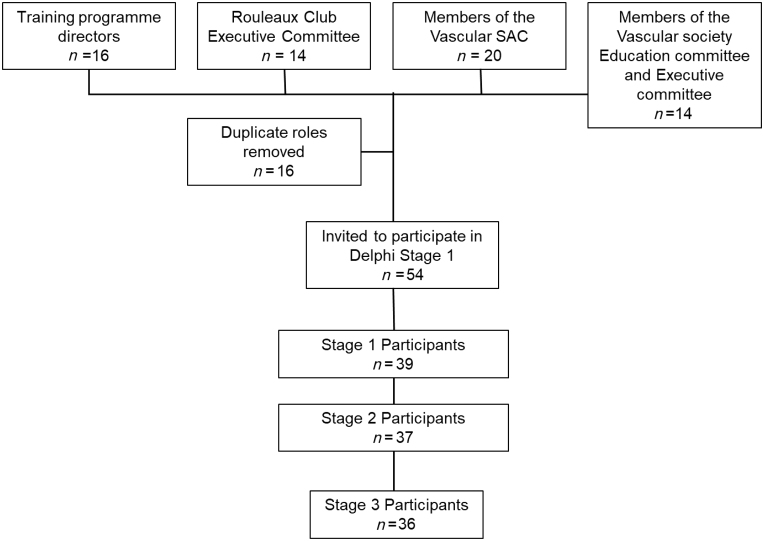
Participants in the three-stage Delphi process

The same group identified stakeholders as experts suitable to invite to participate in the Delphi process. Stakeholders were defined as those directly involved in participating in, delivering or designing the training programme. The stakeholder groups were deemed to be trainees (The Rouleaux Club committee members were utilised, all elected roles voted for by trainees to represent them), training programme directors, members of the vascular SAC, Vascular Society (VS) education committee and VS executive committee (Figure 1). These were all appointed or elected roles.

### Delphi consensus

Stage 1 – Participants were asked to rate how appropriate they perceived each domain to be for assessing the quality of a vascular training programme, using a nine-point Likert scale, ranging from one (inappropriate) to nine (extremely appropriate). Domain headings were given with a brief explanation of what would be included and assessed in that domain. In this stage, participants were invited to suggest additional domains not already presented in Stage 1.

Stage 2 – All participants from Stage 1 were invited to participate in Stage 2. Descriptive statistics from the responses obtained in Stage 1 were included to allow participants to understand the initial degree of consensus. Domains were organised into four groups according to summary scores: ‘consensus appropriate’, ‘consensus inappropriate’, ‘disagreement’ and ‘equivocal’ (see *Data analysis*). No further response was solicited for ‘consensus inappropriate’ definitions.

Stage 3 – All participants from Stage 1 were invited to participate in Stage 3. Descriptive statistics from the responses obtained in Stage 2 were presented (see *Data analysis*). Participants were asked to rate, on a nine-point Likert scale, how strongly they agreed that each of the ‘consensus appropriate’ domains should be used in the QA process and how strongly they agree that each of the ‘consensus inappropriate’ domains need not be used in the quality assessment of a training programme. Participants were also asked to prioritise the domains in order of importance (1 being the most important) for assessing the quality of the training programme.

### Data analysis

#### Analysis of Delphi Stage 1

Descriptive statistics were used to summarise the results of Stage 1, including the number of participants rating the outcome as either seven, eight or nine on the Likert scale (very appropriate/extremely appropriate). Domains rated seven, eight or nine on the Likert scale by at least 20 percent of participants were retained for the next stage. All other domains were discarded.

#### Analysis of Delphi Stage 2

For each domain presented in Stage 2 the proportion of participants scoring 1–3, 4–6 and 7–9 on the nine-point Likert scale was calculated. For the purpose of the Delphi process, ‘appropriate’ referred to appropriateness to assess the quality of the vascular training programme. ‘Consensus appropriate’ was defined as greater than 70 percent of items scoring 7–9 and less than 25 percent of participants scoring 1–3. ‘Consensus inappropriate’ was defined as greater than 70 percent of participants scoring 1–3 AND less than 25 percent of participants scoring 7–9. ‘Disagreement’ occurred when 33 percent or more scored 1–3 and 33 percent or more scored 7–9 for a particular domain. All other combinations were considered ‘Equivocal’. All domains were designated into one of these four categories. ‘Consensus appropriate’ items were brought forward for the third stage and ‘Consensus inappropriate’ and ‘Equivocal’ items were discarded.

#### Analysis of Delphi Stage 3

For each domain presented in Stage 3 the proportion of participants scoring 1–3, 4–6 and 7–9 on the nine-point Likert scale were calculated. The definitions of consensus described in round 2 was applied to these data.

Domains designated ‘Consensus appropriate’ were defined in the final report as appropriate to assess the quality of the vascular training programme. Any remaining domains designated ‘Disagreement’ were discussed at the final consensus meeting of stakeholders. All other domains were discarded.

#### Rationalisation of domains

To confirm consensus and rationalise assessment to ten domains the average prioritisation score of each domain was calculated as well as the proportion of participants ranking each domain in the top ten most important.

## Results

A total of 54 vascular surgeons and vascular trainees were invited to participate in the Delphi consensus; 39 responded to stage 1 (25 consultants and 14 trainees). Of these, 15 were members of the SAC (1 trainee, 14 consultants), 11 were TPDs (5 of whom also sit on the SAC) and 10 were members of the VS executive committee or education committee (1 of whom was also a TPD, 3 were on the SAC and 1 was a trainee). Stages 2 and 3 received 37 and 36 responses, respectively (Figure 1).

### Stage 1

In all, 20 domains were entered into the Delphi process (Table 1). The mean Likert score of all domains was 7.5 (standard deviation (SD) 0.58). All domains were deemed consensus appropriate. The mean Likert score for each domain ranged from 6.5 to 8.3 (SD 0.57–1.88). Table 2 summarises the domains accepted and rejected through each stage of the Delphi process; 16 additional domains were suggested by the experts, 9 of which were already encompassed within the existing domains and so were rejected by the QA group for Stage 2 (Table 3); 7 were included in Stage 2.

**Table 2:** Delphi process results

**Original domains**	**Consensus appropriate mean Likert score**			
	**Stage 1 (percentage Likert ≥7)**	**Stage 2 (percentage Likert ≥7)**	**Stage 3 (Percentage Likert ≥7)**	**Accepted prioritisation score (average prioritisation score)**
JCST survey	**Yes 6.8 (72%)**	**Yes 6.6 (73%)**	**Yes 6.6 (72%)**	**Yes 7 (9)**
GMC survey	**Yes 6.5 (67%)**	No 5.7 (43%)	–	–
Clinical experience	**Yes 8.7 (100%)**	**Yes 8.6 (97%)**	**Yes 8.3 (94%)**	**Yes 1 (1)**
Subspecialty training opportunities	**Yes 7.4 (82%)**	No 6.7 (65%)	–	–
Educational programmes	**Yes 8.3 (97%)**	**Yes 8.2 (92%)**	**Yes 7.7 (92%)**	**Yes 3 (6)**
Trainer support of trainees	**Yes 8.3 (97%)**	**Yes 8.6 (100%)**	**Yes 7.9 (89%)**	**Yes 7 (9)**
Radiology support	**Yes 8.1 (95%)**	**Yes 7.8 (92%)**	**Yes 7.3 (78%)**	**Yes 7 (9)**
Opportunities for professional development	**Yes 7.7 (90%)**	**Yes 7.8 (95%)**	**Yes 7.2 (75%)**	**Yes 6 (8)**
Access to and provision of simulation training	**Yes 7.7 (85%)**	**Yes 7.4 (75%)**	No 6.7 (56%)	No 16 (11)
Research opportunities and output	**Yes 7.0 (69%)**	No 6.6 (64%)	–	–
ARCP outcomes	**Yes 7.6 (87%**)	**Yes 7.8 (89%)**	No 6.9 (69%)	No 11 (10)
Training post information and administration	**Yes 7.5 (82%)**	**Yes 7.5 (92%)**	No 6.6 (53%)	No 13 (12)
Regional support for trainees	**Yes 7.9 (92%)**	**Yes 8.1 (89%)**	**Yes 7.4 (78%)**	**Yes16 (11)**
WBAs	**Yes 6.7 (62%)**	No 6.6. (69%)	–	–
Timetable	**Yes 7.5 (87%)**	**Yes 7.9 (97%)**	**Yes 7.6 (83%)**	**Yes 7 (9)**
Completion of checklists	**Yes 7.1 (77%)**	**Yes 6.6 (72%)**	No 6.5 (56%)	No 13 (12)
FRCS (Vasc) exam results	**Yes 7.4 (72%)**	**Yes 7.6 (86%**)	No 7.2 (69%)	No 11 (10)
Trainee recommendation of the post as per JCST survey	**Yes 7.5 (90%)**	**Yes 7.8 (94%)**	**Yes 7.4 (83%)**	**Yes 7 (9)**
Trainee rated quality of consultant teaching and training as per JCST survey	**Yes 7.6 (95%)**	**Yes 7.6 (97%)**	**Yes 7.5 (83%)**	**Yes 4 (7)**
Quality of AES feedback report	**Yes 6.8 (67%)**	**Yes 6.6 (72%)**	No 6.8 (56%)	No 13 (12)
**Additional Suggested domains**				
Joy at work	–	No 6.6 (64%)	–	–
Whether trainers have designated time within their job plans to act as AES	–	No 6.8 (67%)	–	–
Consultant jobs at the end of the programme. Need for specialist fellowship, e.g. endovascular	–	No 6.2 (53%)	–	–
NVR logged procedures	–	No 6.3 (64%)	–	–
Dropout rate of trainees, particularly important at ST1 and 2 when they start	–	No 6.6 (58%)	–	–
The colleague test: i.e. would your colleagues let you operate on their relative. Would you be happy to have this person as a colleague? Do you think they can do the job?	–	No 6.3 (58%)	–	–
Educational qualifications of consultants in the units	–	No 4.9 (22%)	–	–

AES = assigned educational supervisor; ARCP = annual review of competence progression; FRCS = Fellowship of the Royal Colleges of Surgeons; GMC = General Medical Council; JCST = Joint Committee for Surgical Training; NVR = National Vascular Registry; WBA = work based assessments

**Table 3:** Suggested domains excluded as already incorporated into another domain

**Suggested domain**	**Existing domain covering suggestion**
Mentorship opportunities, who to contact, what support offered	Regional support for trainees
Access to regular interventional radiology lists	Radiology support
Structured work schedules, LFG frequency and multidisciplinary unit attendance, IRMER compliance, access to peripheral angiography lists, team training	Timetable
Split site working, i.e. at both arterial and nonarterial centres	Clinical experience
Quality and frequency of regional teaching programme	Educational programme
Number of index cases undertaken in each individual unit - it is impossible to train if the unit is not undertaking the cases	Clinical experience
Leadership training	Access to simulation
Time spent in each unit and how often rotations change	Training programme information
Destination for senior trainees – consultant jobs/need for fellowship	Suggested twice

IRMER = ionising radiation (medical exposure) regulations; LFG = local faculty group

### Stage 2

A total of 27 domains were entered into Stage 2; 16 were deemed consensus appropriate for stage 3 (mean Likert score range 6.6–8.6, SD 0.49–1.96). Domains with mean Likert scores of 4.9–6.8 were removed (11 domains). These included work-place based assessments (mean Likert score 6.6, SD 1.98), research opportunities (mean Likert score 6.6, SD 1.58), GMC survey (mean Likert score 5.7, SD 1.84), subspeciality training opportunities (mean Likert score 6.7, SD 1.31), and all the newly suggested domains from Stage 1. Table 2 shows the domains retained for Stage 3.

### Stage 3

A total of 16 domains entered stage 3 of the Delphi process (Table 2); 10 were deemed consensus appropriate (mean Likert score range 6.6–8.3, SD 0.84–1.48). These domains were JCST survey results, clinical experience, educational programme, radiology support, timetable, regional support for trainees, trainer support for trainees, opportunities for professional development, trainee-rated quality of consultant teaching and training and trainee recommendation of the post. Six domains with mean Likert scores of 6.47–7.17 (SD 1.34–1.64) were discarded: access to and provision of simulation training (mean Likert score 6.7, SD 1.39), annual review of competence progression (ARCP) outcomes (mean Likert score 6.9, SD 1.64), training postinformation (mean Likert score 6.6, SD 1.36), quality of assigned educational supervisor (AES) feedback report (mean Likert score 6.8, SD 1.13), completion of checklists (mean Likert score 6.5, SD 1.55), and Fellowship of the Royal Colleges of Surgeons (FRCS) exam results (mean Likert score 7.2, SD 1.34). Table 3 shows the final domains deemed consensus appropriate.

### Rationalisation to ten priority domains

Altogether, ten domains were deemed consensus appropriate. The prioritisation score of the domains was calculated according to the study protocol. All ten that were identified as suitable to use in programme QA were ranked in the top ten by more than 50% of Delphi participants, and had an average prioritisation score greater than or equal to nine.

## Discussion

Assessing quality involves making a comparison of one thing against other things of a similar kind. However, definitions of quality are ultimately ‘constructed in the minds of the definers’. ^6^

The Delphi technique is a method for organising conflicting values and experiences, which facilitates the incorporation of multiple opinions into consensus decisions. It is used to combine expert opinion systematically to arrive at an informed group consensus on a complex problem.^7^

Surgical training programmes are complex and involve a broad spectrum of stakeholders, with varied backgrounds. This presents risk of variation in views and priorities or what constitutes ‘quality’ in a training programme, making Delphi methodology highly appropriate.^7^

The GMC defines QA and standards for the management and delivery of undergraduate and postgraduate medical education and training. The JCST also has clearly defined quality indicators for each surgical specialty training programme.^8^ Quality processes follow these principles but, although carefully considered and extensive, they do not cover all aspects that trainees, TPDs and other parties involved in the delivery of the training programme consider as adding significant value to vascular surgical training.

Using Delphi methodology, this study sought to identify the most important domains of vascular surgical training for inclusion in training programme quality assessment and QA. The principal finding was the prioritisation of ten domains deemed appropriate for programme assessment by key stakeholders. As far as the authors are aware this is the first time a Delphi process has been employed to establish consensus on the quality assessment of training programmes.

The ten domains that were deemed consensus appropriate to assess the quality of a training programme are all considered existing markers of quality.

### Training programme survey

The JCST survey has been a compulsory component of all UK surgical training programmes since 2011.^9^ It was developed by JCST in conjunction with the various Schools of Surgery in the UK, in an attempt to limit the number of surveys required from trainees.^10^ Its strengths include the achievement of this first objective, now representing the single compulsory training survey for most UK surgical trainees. It is also described as anonymous, although supervisors and various training body officials can access an individual’s responses, which presents a risk of under-reporting. It is placement-specific, meaning that answers relate to a training post, rather than a training centre, although this does mean completion might be required biannually. However, as each individual training post is usually undertaken by just one or two trainees per year, there is a risk that small numbers of responses may not accurately reflect the quality of the training post, or that individual circumstances may make responses nonrepresentative. There is, therefore, a need to consider multiple responses in the longitudinal assessment of any training post and, with a relatively small number of vascular surgery trainees in each Deanery, the same principle applies. An unclear range of factors also seems to substantially influence the responses to the JCST survey, as exemplified in a comparison of multiple training surveys in 2017,^9^ which demonstrated very poor correlation between surveys, leading to widely varying quality ranking across centres, depending which survey was examined.

### Clinical experience

The JCST Quality Indicators (QI) for vascular surgery^8^ have four QIs relating to clinical exposure. Clinical exposure is required to achieve the competencies dictated by the curriculum^11^ and completion of these competencies is required to obtain Certificate of Completion of Training (CCT) and recommendation to join the GMC specialist register. This is ultimately the entire purpose of the training programme; to deliver the curriculum to produce ‘competent doctors able to deliver excellent outcomes for patients as consultant vascular surgeons’.^11^

### Educational programme

Formal teaching is another QI from the JCST but is also an integral part to gaining knowledge and the evidence base for current practice. The ASPIRE (Annual SPecIalist Registrar Educational) programme is unique to Vascular Surgery and consists of specific educational days for each year of specialty training. Those who have attended the ASPIRE 7 course are historically more likely to pass the FRCS(Vasc) exam than those who have not. Both local and national educational programmes contribute to trainees gaining competencies and knowledge to enable them to pass the FRCS(Vasc) exam and achieve eligibility for completion of training.

### Radiology support

Endovascular intervention is important for the management of multiple vascular pathologies and the curriculum strives to deliver surgeons who can perform hybrid revascularisation procedures and perform endovascular interventions in collaboration with interventional radiologists (IR). Cross-specialty training provides opportunities to optimise experience and exposure to a variety of techniques for both IR and vascular trainees. Collaboration between the specialities is fundamental to the successful delivery of the curriculum.

### Regional support for trainees

The Junior Doctors Contract (2016) identified a range of factors that were having a significant impact on the quality of life of doctors in training. These included geographical limitations of training, limited opportunities for doctors to train flexibly, varying equity in study budget provision and inequality for those who take time out of training.^12^ Consequently ‘Enhancing Junior Doctors’ Working Lives’ programme was established and, as part of this, the SuppoRTT (Supported Return to Training) programme was developed. The 2019 Rouleaux Club survey (submitted but not yet in print) investigated the reasons why trainees left vascular surgical training. Of trainees appointed to the national training programme between 2013 and 2019, 15.4% resigned (unpublished data). Support for trainees directly impacts on their health and wellbeing and their subsequent retention in the training programme. The JCST also recognises the need for supportive processes in its QI.

### Trainer support for trainees

The JCST QIs clearly indicate that consultant supervision must be appropriate for trainees’ level of training and experience and supervised activities should include theatre, clinic, ward rounds and emergency activity.

### Opportunities for professional development

Both the vascular curriculum and JCST QI feature aspects of professional development. Attendance at conferences is key to staying up to date as well as enhancing research and quality improvement. Attendance at courses allows for the acquisition of new skills and specialist knowledge that complements local training resources.

### Strengths and weaknesses

Strengths of this study included the participation of key stakeholders in vascular surgical training, who are ideally placed to determine the areas of greatest importance. There was a good response rate (>70%) with wide UK geographical coverage, and effective trainee representation. The inherent strengths of electronic Delphi methodology, such as its low cost, flexibility and reflexivity, a degree of anonymity, convenience for distance participation, and ability to organise widespread and diverse group thinking, proved useful in this study.^7^

Potential limitations include those inherent to electronic Delphi methodology, such as an inability to control potential distractors during participation and possible technology difficulties, although no participant or invited stakeholder reported such difficulties. There was some participant attrition, although with just one participant from Stage 1 failing to complete Stage 3, this is unlikely to have changed the Delphi outcome substantially. As a self-selected group of educationalists, there is a risk that the expert participants held views that differed to the broader vascular surgery professional community.

### Next steps

These domains will be used by the key stakeholders in conjunction with the JCST survey and JCST QIs to develop a scoring system to provide an objective measure of quality of the vascular training programmes across the UK. Data will be collected electronically from LMs, TPDs and trainees (JCST survey). This process will be piloted and evaluated prospectively to accurately establish a scoring system that is transparent, reliable and reproducible, allowing year-on-year comparison of the training programmes.

## Conclusion

This study has identified the most important domains of vascular surgical training for inclusion in training programme quality assessment. These data will permit the development of a scoring system for the formalisation of the annual assessment of vascular training programme quality in the UK, permitting a reliable and reproducible year-on-year comparison of programme performance. The methodology and results of this study are likely to be transferable to other training programmes beyond the UK. If used appropriately, this study might offer greater potential to flag up areas of underperformance and highlight the best performing training programmes to share best practice. Piloting and prospective evaluation will be necessary during implementation of the new system.
